# Drift of the Subgingival Periodontal Microbiome during Chronic Periodontitis in Type 2 Diabetes Mellitus Patients

**DOI:** 10.3390/pathogens10050504

**Published:** 2021-04-22

**Authors:** Irina P. Balmasova, Evgenii I. Olekhnovich, Ksenia M. Klimina, Anna A. Korenkova, Maria T. Vakhitova, Elmar A. Babaev, Leyla A. Ovchinnikova, Yakov A. Lomakin, Ivan V. Smirnov, Victor N. Tsarev, Ashot M. Mkrtumyan, Alexey A. Belogurov, Alexander G. Gabibov, Elena N. Ilina, Sergey D. Arutyunov

**Affiliations:** 1 Ministry of Healthcare of the Russian Federation, A.I. Evdokimov Moscow State University of Medicine and Dentistry, 127473 Moscow, Russia; iri.balm@mail.ru (I.P.B.); babayev_elmar@mail.ru (E.A.B.); TSAREV_VN@msmsu.ru (V.N.T.); vagrashot@mail.ru (A.M.M.); 2 Federal Research and Clinical Center of Physical and Chemical Medicine, Federal Medical and Biological Agency of Russia, 119435 Moscow, Russia; jeniaole01@gmail.com (E.I.O.); ppp843@yandex.ru (K.M.K.); korenkovaa@mail.ru (A.A.K.); prostonazvanie@mail.ru (M.T.V.); 3 Shemyakin-Ovchinnikov Institute of Bioorganic Chemistry RAS, 117997 Moscow, Russia; leyla_ovchinnikova@yahoo.com (L.A.O.); yasha.l@bk.ru (Y.A.L.); smirnov@mx.ibch.ru (I.V.S.); belogurov@mx.ibch.ru (A.A.B.J.); 4 Chemistry Department, Lomonosov Moscow State University, 119991 Moscow, Russia; 5 Department of Life Sciences, Higher School of Economics, 101000 Moscow, Russia

**Keywords:** oral microbiome, type 2 diabetes mellitus, periodontitis, 16S rRNA gene sequencing, metagenomics

## Abstract

Since periodontitis and type 2 diabetes mellitus are complex diseases, a thorough understanding of their pathogenesis requires knowing the relationship of these pathologies with other disorders and environmental factors. In this study, the representability of the subgingival periodontal microbiome of 46 subjects was studied by 16S rRNA gene sequencing and shotgun sequencing of pooled samples. We examined 15 patients with chronic periodontitis (CP), 15 patients with chronic periodontitis associated with type 2 diabetes mellitus (CPT2DM), and 16 healthy subjects (Control). The severity of generalized chronic periodontitis in both periodontitis groups of patients (CP and CPT2DM) was moderate (stage II). The male to female ratios were approximately equal in each group (22 males and 24 females); the average age of the subjects was 53.9 ± 7.3 and 54.3 ± 7.2 years, respectively. The presence of overweight patients (Body Mass Index (BMI) 30–34.9 kg/m^2^) and patients with class 1–2 obesity (BMI 35–45.9 kg/m^2^) was significantly higher in the CPT2DM group than in patients having only chronic periodontitis or in the Control group. However, there was no statistically significant difference in all clinical indices between the CP and CPT2DM groups. An analysis of the metagenomic data revealed that the alpha diversity in the CPT2DM group was increased compared to that in the CP and Control groups. The microbiome biomarkers associated with experimental groups were evaluated. In both groups of patients with periodontitis, the relative abundance of *Porphyromonadaceae* was increased compared to that in the Control group. The CPT2DM group was characterized by a lower relative abundance of *Streptococcaceae*/*Pasteurellaceae* and a higher abundance of *Leptotrichiaceae* compared to those in the CP and Control groups. Furthermore, the CP and CPT2DM groups differed in terms of the relative abundance of *Veillonellaceae* (which was decreased in the CPT2DM group compared to CP) and *Neisseriaceae* (which was increased in the CPT2DM group compared to CP). In addition, differences in bacterial content were identified by a combination of shotgun sequencing of pooled samples and genome-resolved metagenomics. The results indicate that there are subgingival microbiome-specific features in patients with chronic periodontitis associated with type 2 diabetes mellitus.

## 1. Introduction

Development of the high-throughput sequencing technologies has resulted in a significant breakthrough in human microbiome research. Next-generation sequencing [1] and LC-MS/MS analysis [2,3] have become unique tools in studying the taxonomic and functional characteristics of the human microbiome, as well as the pathological processes associated with it. However, like any breakthrough technology, the resulting data have raised more and more questions yet to be answered [4]. The human oral cavity is one of the most exciting microbial habitats. Over 700 microbial species inhabiting the oral cavity and forming unique communities have been found [5,6,7]. The composition of such communities is relatively stable for the healthy oral cavity [8] and plays an important role in maintaining a dynamic ecological balance with the host [9,10]. The most inhabited part of the oral cavity is the periodontal sulcus, which contains about 400–500 microbial species [9,11]. Changes in the diversity and biological functions of the periodontal sulcus microbiome lead to an altered state, which can be associated with the onset and progression of periodontal diseases [12], as well as other local or systemic pathological conditions [13,14].

One of the most frequent associations of periodontal diseases with systemic pathology is the combination of chronic periodontitis (CP) with type 2 diabetes mellitus (T2DM) [15,16,17] In this case, chronic periodontitis usually progresses rapidly and has a complicated course [18,19]; excessive and synergistic activation of a number of cytokines is one of the reasons for that [20]. Changes in the composition of oral microbiota are among the key manifestations of the association between these diseases [21,22]. Thus, the potential influence of the oral microbiota on the development of T2DM was studied using the periodontal pathogen *Porphyromonas gingivalis*. Induction of experimental periodontitis in mice by oral administration of *P. gingivalis* has a significant effect on the expression level of the *Irs1* and *Sirt1* genes in the host cells, which suppresses the sensitivity of adipocytes and other cells to insulin [23]. The pathogenetic role of *P. gingivalis* in periodontitis complicated by type 2 diabetes mellitus (CPT2DM) can also be fulfilled by direct stimulation of adipocytes by lipopolysaccharides (LPS), leading to the production of adipocytokines and proinflammatory cytokines provoking oxidative stress. It was also reported that *P. gingivalis* LPS induce secretion of angiopoietin-like protein 2 in epithelial cells of the periodontium, which affects angiogenesis and exhibits proinflammatory properties [24]. These changes can contribute to the development of systemic inflammation and are associated with lipid peroxidation, a part of the diabetes mellitus pathogenesis [25]. Therefore, the oral cavity microbiome (including the periodontal pathogenic bacteria) may be involved in the pathogenesis of diabetes mellitus, which makes it a reasonable study object.

Comparative analysis of the subgingival microbiome in patients with CPT2DM is described in the present study. We used 16S rRNA gene sequencing and compositional data analysis (CoDa) to characterize the subgingival periodontal microbiome. Additionally, dental plaque samples were pooled and characterized using shotgun sequencing and genome-resolved metagenomics approaches.

## 2. Material and Methods

### 2.1. Subject Population and Study Design

**Figure 1:**
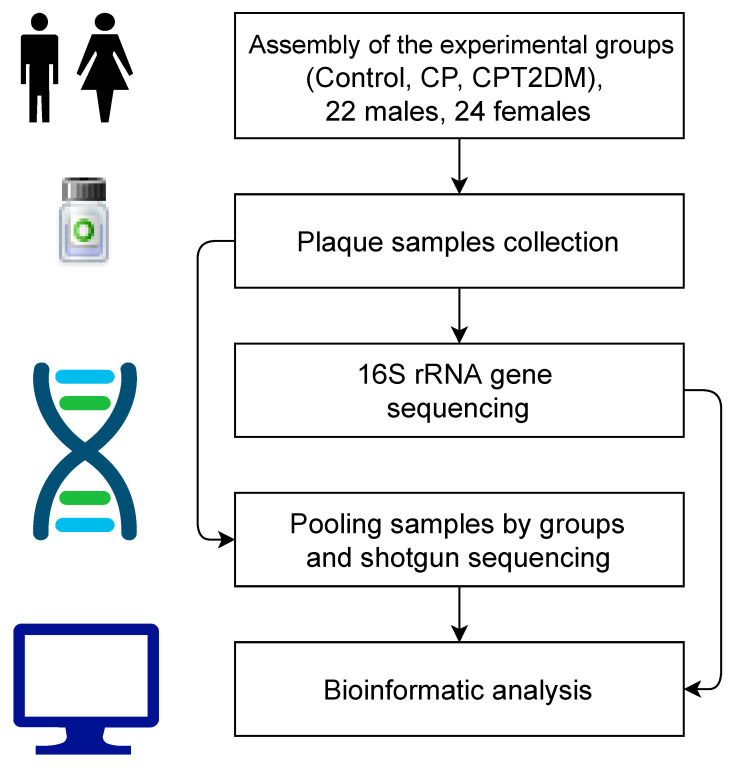
Schematic visualization of the experimental design. CP: chronic periodontitis. CPT2DM: chronic periodontitis with type 2 diabetes mellitus.

The study involved 46 subjects consecutively selected from the population and subdivided into three groups (Table 1). The index group consisted of 15 patients with chronic periodontitis associated with type 2 diabetes mellitus (CPT2DM group); the reference group consisted of 15 patients with chronic periodontitis without somatic comorbidities (CP group), and the control group consisted of 16 subjects with signs of neither chronic periodontitis nor type 2 diabetes mellitus (Control group). Controls were healthy subjects with no history of type 2 diabetes mellitus or smoking. All nondiabetic patients were required to have HbA1c level ≤6.0%. All the subjects were followed up at the clinics of the Department of Propaedeutic Dentistry of the A.I. Evdokimov Moscow State University of Medicine and Dentistry. The study was approved by the University Ethics Commission and was conducted in full compliance with the Declaration of Helsinki, the International Conference on Harmonization’s Good Clinical Practice, and appropriate local legislation. T2DM subjects were treated at the Department of Endocrinology and Diabetology of the same university. Patients had not received treatment for chronic periodontitis over the past 6 months. The schematic visualization of the experimental design is presented in Figure 1.

### 2.2. Diagnostic and Inclusion Criteria

Control donors were added to the study only after consultation with a dentist and an endocrinologist. Patients were diagnosed with chronic periodontitis according to the clinical and radiological data in compliance with the 2018 classification of periodontal diseases [26].

The exclusion criteria were as follows: pregnancy, lactation, use of antibiotic, anti-inflammatory, or immunosuppressive therapies during the previous six months, regular use of mouth rinses containing antimicrobials, use of orthodontic appliances, presence of other systemic conditions that could affect the progression of periodontitis (e.g., immunological disorders or osteoporosis).

The study groups contained approximately equal numbers of non-smoking males and females aged 41–65 years. Periodontitis patients had stage II (moderate) periodontitis, with a generalized lesion, probing pocket depths of 3–4 mm, loss of bone tissue around the teeth no more than 1/3 of the root length, and virtually no tooth loss associated with periodontitis. Patients with type 2 diabetes mellitus were in remission.

The analysis of the dental status included determining the following indices adopted in dental studies: PHP (Patient Hygiene Performance); OHI-S (Simplified Oral Hygiene Index); CAL (Clinical Attachment Level); MMI (Miller’s mobility index); PBI (Papillary Bleeding Index); and TL (Tooth loss). Patients were diagnosed with type 2 diabetes mellitus in accordance with the World Health Organization’s (WHO) diagnostic criteria 1999/2006/2011 [27] with allowance for the clinical, anamnestic, and laboratory evaluations. In all T2DM patients, disease duration was 3 to 7 years; the disease course was moderately compensated; blood glucose level was below 7.8 mmol/L, and glycated hemoglobin level was <8%. All subjects in the CPT2DM group received basic antidiabetic therapy: 7 subjects received insulin therapy, and 8 subjects received oral glucose-lowering drugs.

### 2.3. Collection and Sequencing of Plaque Samples

The contents of the periodontal pocket in patients with chronic periodontitis (CP), chronic periodontitis associated with type 2 diabetes mellitus (CPT2DM) and the contents of the gingival sulcus in control subjects were the study material. The samples were collected from the patients in the morning on an empty stomach (between 9:00 and 11:00 a.m.) before they used a toothbrush and other hygiene products. The biological material was sampled from four spots of the periodontal pockets/sulcus at the level of the second molars using sterile paper endodontic posts, which were placed together into a test tube containing 0.2 mL of sterile physiological saline solution and shaken. Material was collected at six sites per tooth (mesio-, mid-, and disto-buccal area; mesio-, mid-, and disto-lingual area) for all teeth.

The samples were delivered to the laboratory and subsequently stored at −20 °C. Total DNA was extracted from the samples using a QIAamp DNA Investigator Kit (Qiagen, Düsseldorf, Germany) in accordance with the manufacturer’s protocol. Genomic DNA content was determined on a Qubit 2.0 fluorometer (Invitrogen, Carlsbad, CA, USA) in accordance with the manufacturer’s instructions. The enriched microbial DNA (50–100 ng) was fragmented using a Covaris S220 system (Covaris, Woburn, MA, USA). The final fragment size was determined using an Agilent 2100 bioanalyzer (Agilent Technologies, Santa-Clara, CA, USA) in accordance with the manufacturer’s instructions. Briefly, the extracted DNA was amplified using standard 16S rRNA gene primers being complementary to the V3–V4 region and containing 5′-illumina adapter sequences. Sequencing was carried out on a HiSeq 2500 platform (Illumina) in accordance with the manufacturer’s instructions.

DNA samples for shotgun sequencing were pooled and prepared by ligating the genomic DNA of the samples within each study group taken at equimolar amounts. The amount of the mixed DNA pool was determined on a Qubit 2.0 fluorometer (Invitrogen, Carlsbad, CA, USA) in accordance with the manufacturer’s instructions. A NEBnext Microbiome DNA enrichment kit was used for enriching the microbial genomic DNA in the mixed pools in microbial genomic DNA in accordance with the manufacturer’s instructions. The libraries of paired terminal fragments were prepared in accordance with the manufacturer’s guidelines using a NEBNext Ultra II DNA Library Prep Kit (New England Biolabs, Ipswich, MA, USA). The libraries were indexed using NEBNext multiplex Oligos for Illumina kits (96 Index Primers) (New England Biolabs, Ipswich, MA, USA). The distribution over library size and quality was assessed using a high-sensitivity DNA microarray (Agilent Technologies, Santa-Clara, CA, USA). Subsequent quantification of the libraries was performed using a high-sensitivity Quant-iT DNA Assay Kit (Thermo Scientific). Sequencing was conducted on a HiSeq 2500 platform (Illumina) in accordance with the manufacturer’s instructions using the following reagent kits: HiSeq Rapid PE Cluster Kit v2, HiSeq Rapid SBS Kit v2 (500 cycles), HiSeq Rapid PE FlowCell v2, and a 2% Phix spike in controls.

### 2.4. Bioinformatic and Statistical Analysis

The 16S rRNA gene sequencing data were processed using the DADA2 pipeline [28] according to the published protocol [29]. The resulting phyloseq [30] object contained an amplicon sequence variant (ASV) table, a taxonomy table, and a phylogenetic tree. The ASVs were pooled at the family level. In addition, the top eight families in terms of relative abundance were selected for statistical analysis. The CoDa (compositional data analysis) approaches, such as Aitchison distance [31,32] and CoDa dendrogram, were used for data visualization and exploration analysis. The CoDa dendrogram is a dendrogram-like graph that shows: (a) the way of grouping parts of the compositional vector; (b) the explanatory role of each sub-composition generated in the partition process; and (c) decomposition of the total variance into balance components associated with each binary partition [33,34]. Before constructing the CoDa dendrogram, Bayesian estimation of (non-zero) proportions was performed to remove rare taxa and substitute zeros [35]. The Songbird approach [36] implemented in the QIIME2 framework [37] was used to discover biomarkers significantly discriminating the experimental groups. Wilcoxon signed rank test was used for additional statistical comparison. The GNU/R statistical environment was used for data analysis [38].

The metaWRAP pipeline was used for the construction of metagenome-assembled genomes (MAGs) [39] (containing MEGAHIT [40], MetaBAT2 [41], MaxBin2 [42], BWA [43]), with the following parameters of the resulting bins: completeness > 35%, contamination < 15%. Multiple alignment and phylogenetic tree plotting for 43 marker amino acid sequences of MAGs and Human Oral Microbiome Database (HOMD) genomes [44] was performed by CheckM means [45]. The CAT/BAT tool was used for additional taxonomic annotation of MAGs [46]. Next, the closest HOMD genomes to MAGs were found to follow the coverage of the obtained MAGs in all the pooled metagenomic samples.

Statistical analysis of clinical data was performed using the SPSS version 21 software package.

## 3. Results

### 3.1. Description of the Demographic and Clinical Parameters of the Experimental Cohort

The demographic characteristics of the analyzed groups (Table 1) showed no significant intergroup differences in sex and age. However, based on the body mass index parameter, the percentage of overweight patients (Body Mass Index (BMI) 30–34.9 kg/m^2^) and patients with class 1–2 obesity (BMI 35–45.9 kg/m^2^) was significantly higher in the CPT2DM group than in the CP group or in the Control group. Table 2 summarizes the oral health assessed using various dental indices and glycosylated hemoglobin levels. The PHP indices showing the oral hygiene status revealed no differences between the CP and CPT2DM groups but were significantly higher in patients with chronic periodontitis compared to the control group. The CAL indices, which characterize the state of periodontal pockets, were significantly higher in both chronic periodontitis groups (CP and CPT2DM), while there were no changes in other dental status parameters regardless of the association with type 2 diabetes mellitus. The glycosylated hemoglobin (HbA1c) level was expected to be higher in CPT2DM patients, while the blood glucose level was approximately the same in different groups.

Overall, no significant differences between CP and CPT2DM groups were observed for most studied dental indices. Differences between the chronic periodontitis groups identified in subsequent analyses was associated with the presence of type 2 diabetes and not with some other clinical parameters. This allows one to identify significant characteristics of the subgingival microbiota associated with periodontitis and type 2 diabetes.

### 3.2. Characteristic of the Subgingival Periodontal Microbiota Based on 16S rRNA Gene Sequencing of the Collected Samples

**Figure 2:**
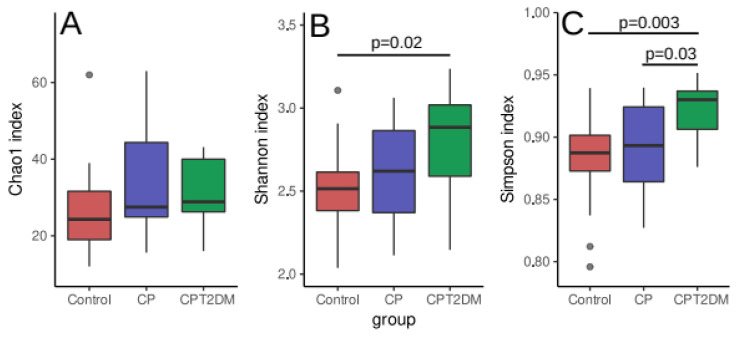
Alpha diversity distribution between the study groups. (**A**) Chao1 index. (**B**) Shannon index. (**C**) Simpson index. The data were analyzed by the Wilcoxon rank-sum test with FDR (false discovery rate) correction for multiple testing. Median, interquartile range and standard deviation are indicated.

The identification of microbiome biodiversity in each clinical group involved two phases. During the first phase, 16S rRNA gene fragments were sequenced to evaluate the abundance of various bacterial families in plaque samples. Data analysis was performed using the DADA2 [28] and phyloseq [30] packages for GNU/R. After quality filtering, the 16S rRNA gene sequencing data contained an average of 50,060 ± 15,902 paired reads per sample. The summary of sequencing statistics is shown in Supplementary Table S1. Statistical intergroup differences in community richness (alpha diversity) were identified using the Chao1, Shannon and Simpson indices. These indices take count of the identified species and their abundance in the microbial community (see Figure 2). An increase in alpha diversity was revealed in the CPT2DM group compared to the Control group using the Shannon index (Wilcoxon rank-sum test with FDR (false discovery rate) correction for multiple testing *p* < 0.05), while the Simpson index showed an increase in statistical significance in the CPT2DM group compared to both the Control and CD groups (Wilcoxon rank-sum test with FDR correction for multiple testing *p* < 0.05). However, no significant intergroup differences in the Chao1 index were revealed.

**Figure 3:**
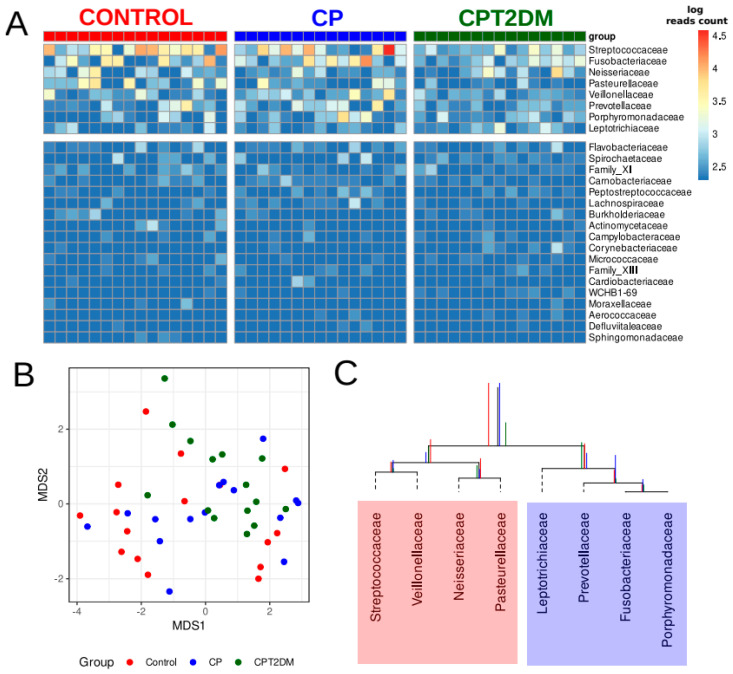
The major bacterial genera present in the subgingival periodontal microbiota of the control and CP/CPT2DM patient groups. (**A**) Columns correspond to the samples; the group is denoted with a top color bar. Hierarchical clustering was performed using the Euclidean distance and complete linkage. Logarithmic transformation of read counts was performed using pseudo counts. The top eight families in terms of relative abundance were selected for further analysis. (**B**) Non-metric multidimensional scaling (NMDS) biplot of taxonomic profiles (family level) of patients’ plaque samples using 16S rRNA gene sequencing and Aitchison distance (MDS1 and MDS2; coordinates scaled to standard deviation unit and centered to the mean). Taxonomic profiles at family level referring to the samples from control subjects, CP, and CPT2DM patients, are shown in red, blue, and green, respectively. (**C**) The CoDa dendrogram shows an ecological model of differences between the experimental groups. Decomposition of total variance by balances between groups of genera is shown using vertical bars (red bars denote the Control group; blue, the CP group; and green, the CPT2DM group). The mean balances are shown using anchoring points of vertical bars. The red area denotes the “healthy” state balance; the blue area denotes the “disease” state balance. CP: chronic periodontitis. CPT2DM: chronic periodontitis with type 2 diabetes mellitus. CoDa: compositional data analysis.

An analysis of the 16S rRNA gene sequencing data revealed 26 bacterial families (Supplementary Table S2) in the samples in all clinical groups. The observed distribution of bacterial families is shown in Figure 3A. The eight most abundant bacterial families were selected for further data analysis. The NMDS (non-metric multidimensional scaling) bidimensional visualization is presented in Figure 3B. The taxonomic data revealed no clustering by groups. The balance dendrogram (CoDa dendrogram) was used to construct the model of taxonomic differences between the experimental groups. This approach allowed us to identify specific balances (the ratio between taxonomic abundances) involved in the distinction between the metagenome groups [33,34]. This model describes the intensity of taxonomic reshapes when the metagenome profile is moving from the “healthy state” to the periodontal “disease state” (see Figure 3C).

According to the CoDa dendrogram, the main balance (denoted as “Balance 1” in Figure 2B) of the CPT2DM group was associated with an increased relative abundance of four bacterial families, such as *Leptotrichiaceae*, *Prevotellaceae*, *Fusobacteriaceae*, and *Porphyromonadaceae*, while the Control group was characterized by increasing relative abundances of *Streptococcaceae*, *Veillonellaceae*, *Neisseriaceae*, and *Pasteurellaceae*. The CP group occupied a boundary position between the CPT2DM and Control groups.

**Figure 4:**
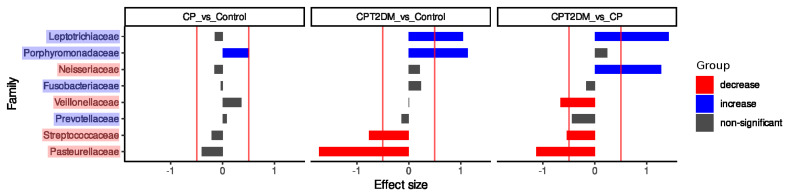
The results of the Songbird analysis. The *X*-axis denotes the effect size; the *Y*-axis denotes the bacterial families. The statistically significant decrease is shown in red; the statistically significant increase is shown in blue.

The Songbird approach was used for statistical validation of the balance dendrogram (Figure 4). The primary output from Songbird is a file containing differentials describing the log-fold change in features with respect to a certain field(s) in sample metadata. The most important aspect of these differentials is rankings, which are obtained by sorting a column of differentials from lowest to highest. These rankings show the information on the relative associations of features with a given covariate [36]. In both groups of periodontitis, the relative abundance of *Porphyromonadaceae* was increased compared to the Control group. The CPT2DM group was characterized by a decreased relative abundance of *Streptococcaceae*/*Pasteurellaceae* and an increased *Leptotrichiaceae* compared to the CP and Control groups. Furthermore, the CP and CPT2DM groups differed in terms of relative abundance of *Veillonellaceae* (decreased in the CPT2DM group compared to CP) and *Neisseriaceae* (increased in the CPT2DM group compared to CP).

### 3.3. Genome-Resolved Metagenomic Analysis of the Shotgun Sequencing Data of the Pooled Samples

At the next stage, the DNA samples within each group were pooled and sequenced using shotgun technology. The summary sequencing statistics are presented in Supplementary Table S1. The bacterial genomes were restored from the metagenomic data using genome-resolved metagenomic approaches based on the metagenomic assembly and clustering of contigs through the metagenomic binning procedure (see Section 2). As a result, 26 MAGs (metagenome-assembled genomes) were assembled for all the metagenomic samples with selected quality parameters (completeness > 35%, contamination < 15%). Six MAGs were obtained for the Control group; 9 and 11 MAGs, for the CP and CPT2DM groups, respectively. The binning statistics are shown in Supplementary Table S3.

**Figure 5:**
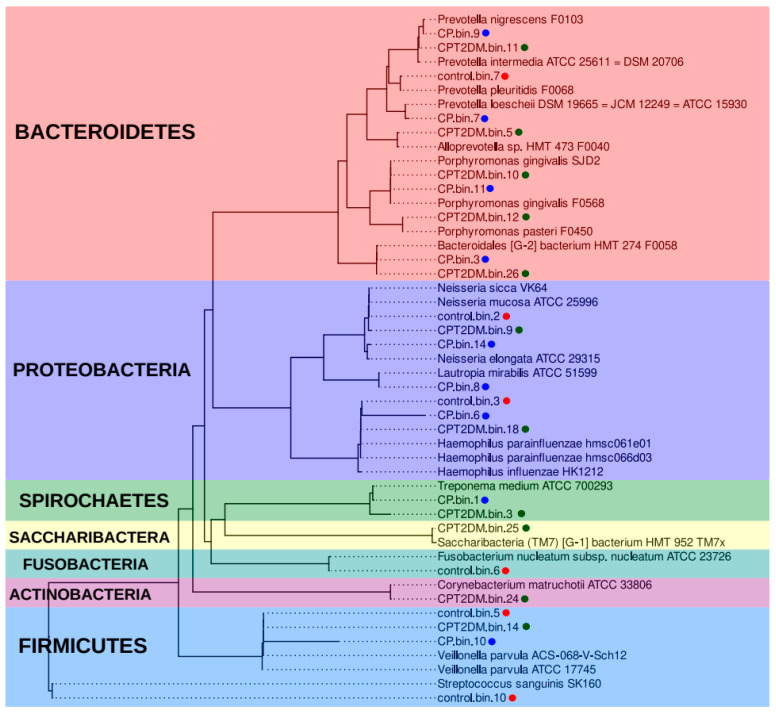
Phylogenetic analysis of the metagenome-assembled genomes. The phylogenetic tree is based on 43 marker proteins obtained from 26 MAG sequences and related eHOMD genomes [44]. The MAG groups are shown in different colors (red for the Control group, blue and green for CP and CPT2DM, respectively). The colored areas also denote the bacterial phyla. MAG: metagenome-assembled genome. eHOMD: expanded Human Oral Microbiome Database.

The analysis of MAGs involved two steps. First, taxonomic annotation of MAG sequences was obtained using the CAT/BAT tool (see Supplementary Table S3). A unified multiple alignment and a phylogenetic tree (also using the CheckM tool) were constructed using MAGs sequences and expanded Human Oral Microbiome Database (eHOMD) bacterial genomes [44] to verify the taxonomic annotation. The nearest neighbor MAGs were determined and further used for constructing a combined phylogenetic tree (Figure 5).

According to this analysis, *Haemophilus* spp., *Veilonella* spp., and *Neisseria* spp. bacteria are common in all the analyzed groups. The sample from the Control group is characterized by the presence of unique MAGs close to such bacteria as *Streptococcus sanguinis*, *Fusobacterium nucleatum*, and *Prevotella pleuritis*. Bacteria close to *Lautopia mirabilis*, *Prevotella loescheii*, and *Prevotella nigrescens* were present in the CP MAG group. Bacteria close to *Alloprevotella sp.* HMT 473, *Corynebacterium matruchotii*, *Prevotella intermedia*, *Porphyromonas pasteri*, and *Saccharibacteria* (TM7) [G-1] HMT-952 were present in the CPT2DM MAG group only. Meanwhile, MAGs close to *Porphyromonas gingivalis* and *Bacteroidales* [G-2] HMT-274, as well as *Treponema* spp. (while maintaining species differences), are common for the CP and CPT2DM groups. Therefore, the taxonomic representation in control subjects differs from that in both groups of patients with periodontitis. *Bacteroidetes* and *Spirochaetes* spp. MAGs were found in both groups of periodontitis patients, whereas unique MAGs can also be distinguished between these groups.

## 4. Discussion

Chronic periodontitis (CP) and type 2 diabetes mellitus (T2DM) are widespread multifactorial diseases, which are evidently interrelated [47]. T2DM is one of the main risk factors for the development of periodontitis, while periodontitis severity can affect glycemic control and complications in patients with diabetes (impaired tissue repair capacity being among the reasons for that) [47,48]. Therefore, treatment of periodontitis is considered to be among the beneficial approaches of diabetes therapy [49,50,51]. The consortia of oral bacteria form fairly stable communities [52]. Even in healthy people, the microbial composition of different parts of the oral cavity has its individual characteristics [8].

The disturbances in the gut microbiota have been found to be interrelated with an increased incidence of type 2 diabetes mellitus. Thus, a reduced ratio of bacterial types Bacteroidetes/Furmicutes and a significant reduction in the content of some functionally important bacteria (e.g., Bifidobacterium) in the gut of patients with type 2 diabetes mellitus have been revealed [53]. The development of type 2 diabetes mellitus in humans was reported to be associated with a lower abundance of butyrate-producing bacterial species and increased abundance of Lactobacilla [54,55]. An increased number of some endotoxin-producing Gram-negative bacteria was noted [54,56,57], which alters the energy metabolism of the host and enhances inflammation response [54,58,59]. Disturbances in energy homeostasis lead to hyperglycemia and hyperlipidemia, which can trigger obesity and, ultimately, insulin resistance [60]. It should also be considered that host genetics affect the profile of the gut microbiome, thus ensuring the resilience of this ecosystem [61].

Much less is known about the relationship between the oral microbiome and type 2 diabetes mellitus, although one of the leading mechanisms of the influence of periodontal pathogens on the development and course of diabetes mellitus is associated with the inclusion of these bacteria in the gut microflora [23]. For example, experimental injection of *P. gingivalis* into the oral cavity led to gut colonization with this periodontopathogen that affects glucose metabolism [62,63] by increasing expression of the G6pc gene, which positively regulates gluconeogenesis and increases glucose level [64].

Nonetheless, it is still not exactly clear which oral microorganisms are susceptible to developing diabetes and how exactly diabetes affects them. Griffen et al. [65] described 25 taxa, including six bacterial genera (*Neisseria*, *Streptococcus*, *Haemophilus*, and *Pseudomonas* being among them) whose relative abundance in the oral microbiota differed for patients with T2DM and the control groups. According to previously published [21] and [66], the changes in oral microbiota in patients with T2DM and periodontitis depend both on the glycemic status and stage of the periodontal disease. However, it is still enigmatic which of these factors had the main impact. Wolcott et al. [67] inferred that the observed changes in the subgingival microbiome associated with T2DM and periodontitis are potentially caused by metabolic and immune dysregulation of the host.

In this study, we identified differences in the composition of the subgingival microbiota between groups of control subjects, patients with chronic periodontitis (CP group) and patients with chronic periodontitis associated with type 2 diabetes (CPT2DM group), which can be regarded as potential microbial biomarkers of these pathogenic conditions. The demographic characteristics of the analyzed groups show no significant intergroup differences except for BMI. The percentage of overweight patients (BMI 30–34.9 kg/m^2^) and patients with class 1–2 obesity (BMI 35–45.9 kg/m^2^) was significantly higher in the CPT2DM group than in the CP group or the Control group. This is a significant limitation of the conclusions drawn in our study since the oral microbiome may change in obese people regardless of their glycemic status [68]. No statistically significant difference in the main dental indices was revealed between the CP and CPT2DM groups. However, the glycosylated hemoglobin (HbA1c) level was expectedly higher in the CPT2DM group, while the blood glucose level was approximately the same in different groups.

Alpha diversity was increased in the CPT2DM group compared to the Control and CP groups, while the CP and Control groups did not differ in terms of this parameter. Increased bacterial richness in the oral microbiome is significantly associated with poor oral health, including the presence of decayed teeth, periodontitis, and poor oral hygiene [69]. Earlier, no increased richness of the subgingival community was found in patients with periodontitis [65,70]. The presence of bleeding was not associated with different alpha diversities in patients with periodontitis. However, bleeding sites showed a higher total bacterial load [70].

The differences in microbial content between the tested groups were also discovered. First, an ecological model based on the principles of the compositional data analysis (CoDa) describing the shifts from the “healthy state” to the CPT2DM state was obtained. The findings allowed us to distinguish two different “microbiota states” associated with the Control and CPT2DM groups. The “healthy state” included bacterial families, such as *Streptococcaceae*, *Veillonellaceae*, *Neisseriaceae*, and *Pasteurellaceae*, while *Leptotrichiaceae*, *Prevotellaceae*, *Fusobacteriaceae*, and *Porphyromonadaceae* formed the “disease state.” Interestingly, the CP group occupies a boundary position between the CPT2DM and Control groups. The “disease state” is formed by bacterial families including a wide range of bacteria overrepresented in periodontitis compared to healthy controls [71,72,73,74,75,76], while the “healthy state” is characterized by the formation of the oral microbiota commonly present in healthy subjects [71,73,74,75,76] Second, statistically significant biomarkers distinguishing experimental groups were identified. Both periodontitis groups were associated with an increased relative abundance of *Porphyromonadaceae* compared to healthy controls. However, the CPT2DM group was characterized by a reduced relative abundance of *Streptococcaceae*/*Pasteurellaceae* and increased relative abundance of *Leptotrichiaceae* compared to those in the CP and Control groups. Furthermore, the CP and CPT2DM groups differed in terms of relative abundance of *Veillonellaceae* (decreased in the CPT2DM group compared to CP) and *Neisseriaceae* (increased in the CPT2DM group compared to CP). These findings are partially consistent with the aforementioned ecological model. It was reported previously that the content of pathogenic species was higher in patients with T2DM, both complicated and uncomplicated by periodontitis, compared with the nondiabetic controls [22].

Additionally, the genome-resolved metagenomic methods were used to analyze pooled metagenomic samples. These computational techniques allowed us to reconstruct the bacterial genomes from the metagenomic data (metagenome-assembled genomes, MAGs). We showed that *Haemophilus* spp., *Veilonella* spp., and *Neisseria* spp. MAGs were common in all groups. The main part of *Bacteroidetes* and all the identified *Spirochaetes* MAGs were found in both groups of periodontitis patients, whereas unique MAGs were also present in these groups. Our findings are consistent with the results of previous studies reporting 16S rRNA gene sequencing of subgingival bacterial communities [65]. The bacteria close to *P. gingivalis* and *Bacteroidales* [G-2] bacteria HMT-274, as well as *Treponema medium* (while maintaining species differences), were common for both periodontitis groups. *P. gingivalis* are strongly associated with periodontal disease. It should be emphasized that differences in the revealed *Treponema* species between the CP and CPT2DM groups may be caused by the nature of coaggregation, clinical manifestations of periodontitis, or specific characteristics of the environment.

The differences in the content of *Prevotella* spp. are particularly interesting. Bacteria close to *P. intermedia* were detected only in the pooled metagenomic sample of the CPT2DM group, while *P. nigrescens* and *P. loescheii* were detected only in the CP group. The association of these bacteria with disease severity was previously noted. *P. intermedia* is associated with more severe forms of periodontitis, while *P. nigrescens* is associated with mild to moderate disease [77]. Another distinctive feature of the CPT2DM group was the presence of unique MAGs, namely, *Actinobacteria* (*Corynebacterium matruchotii*) and Candidatus *Saccharibacteria* (TM7) [G-1] HMT-952. *Corynebacterium matruchotii* was implicated in the nucleation of oral microbial consortia leading to biofilm formation [78]. The role played by TM7x bacteria in the oral microbiome has yet to be elucidated. It is worth noting that the pooled metagenomes are not sufficiently representative to determine any significant differences in the experimental groups. Nevertheless, the results are consistent and complement the findings of the 16S rRNA gene sequencing analysis.

## 5. Conclusions

The taxonomic composition of the subgingival microbiome clearly differentiates between the “healthy state” and the “disease state”, as well as during a possible transition to chronic inflammation associated with T2DM. Importantly, this process is accompanied by increased microbiome biodiversity. The identified biomarkers of the analyzed clinical patterns may be further utilized for developing test systems to be used in routine clinical practice.

## Figures and Tables

**Table 1:** Demographic and clinical parameters of chronic periodontitis patients with and without type 2 diabetes mellitus and control donors.

Parameters		Study Group (Subjects)			One-Way ANOVA	
		CPT2DM (n = 15)	CP (n = 15)	Control (n = 16)	F	*p*
Sex	Male	7	7	8	0.080	0.786
	Female	8	8	8		
Age	Male	57.8 ± 6.3	55.7 ± 9.6	48.2 ± 5.7	2.739	0.142
	Female	57.9 ± 6.4	56.2 ± 8.6	48.9 ± 6.7		
BMI *	25.0–29.9 kg/m^2^	4 *	12	10	5.942	0.019 *
	30.0–34.9 kg/m^2^	5 *	3	5		
	35.0–45.9 kg/m^2^	6 *	0	1		

* BMI—Body Mass Index; CP: chronic periodontitis; CPT2DM: chronic periodontitis with type 2 diabetes mellitus; significantly differing population distribution according to BMI was observed in CPT2DM patients compared to control subjects and patients with CP only (* *p* < 0.05).

**Table 2:** Comparison of study groups using dental status indices and the glycosylated hemoglobin level.

Variable		Study Group, Median (Min; Max)			Wilcoxon Rank Sum Test *p*-Value
		CPT2DM (n = 15)	CP (n = 15)	Control (n = 16)	
Dental status indices	PHP	0.7 (0; 2.1)	1.3 (0.6; 2.1)	0.2 (0; 0.7)	*p*_(CP2DM-CP)_ = 0.234 *p*_(CPT2DM-Control)_ = 0.085 *p*_(CP-Control)_ < 0.010 *
	OHI-S	1.4 (0.4; 4.5)	1.4 (0.4; 3.3)	1.8 (0.5; 3.1)	*p*_(CP2DM-CP)_ = 0.718 *p*_(CPT2DM-Control)_ = 0.627 *p*_(CP-Control)_ = 0.958
	CAL	3.9 (3.0; 4.5)	3.8 (3.4; 4.3)	0.8 (0; 1.1)	*p*_(CP2DM-CP)_ = 0.697 *p*_(CPT2DM-Control)_ < 0.001 * p_(CP-Control)_ < 0.001 *
	MMI	0.8 (0; 2.0)	0.5 (0; 2.0)	0 (0; 1.0)	*p*_(CP2DM-CP)_ = 0.409 *p*_(CPT2DM-Control)_ = 0.056 *p*_(CP-Control)_ = 0.122
	TL	0 (0; 0)	0.05 (0; 1.0)	0 (0; 0)	*p*_(CP2DM-CP)_ = 0.697 *p*_(CPT2DM-Control)_ = 0.998 *p*_(CP-Control)_ = 0.874
	PBI	0.34 (0; 0.90)	0.30 (0.10; 0.90)	0.38 (0; 0.84)	*p*_(CP2DM-CP)_ = 0.697 *p*_(CPT2DM-Control)_ = 0.584 *p*_(CP-Control)_ = 0.874
Diabetes criteria	Glucose (mmol/L)	5.7 (5.0; 7.8)	5.6 (5.1; 6.1)	5.4 (4.4; 6.6)	*p*_(CP2DM-CP)_ = 0.748 *p*_(CPT2DM-Control)_ = 0.665 *p*_(CP-Control)_ = 0.589
	Glycosylated hemoglobin (HbA1c)	7.5 (6.5; 8.0)	4.5 (3.0; 6.0)	3.5 (3.0; 5.0)	*p*_(CP2DM-CP)_ < 0.001 * p_(CPT2DM-Control)_ < 0.001 * p_(CP-Control)_ = 0.067

PHP: patient hygiene performance. OHI-S: simplified oral hygiene index. CAL: clinical attachment level. MMI: Miller’s mobility index. TL: tooth loss. PBI: papillary bleeding index; The distributions of investigated parameters analyzed by Wilcoxon rank sum test. * *p* < 0.05.

## Data Availability

The analytical scripts and obtained data are available at https://github.com/RCPCM-GCB/CPT2DM_project (accessed on 13 September 2020). Raw 16S rRNA gene sequencing data are also deposited at the NCBI Sequence Read Archives under the BioProjects accession number PRJNA664107. Data available in a publicly accessible repository https://www.ncbi.nlm.nih.gov/bioproject/PRJNA664107.

## Supplementary Materials

The following are available online at https://www.mdpi.com/article/10.3390/pathogens10050504/s1, Table S1. Sequencing quality control data. Table S2. Taxonomic composition of 16S rRNA gene sequencing samples at the family level (reads counts). Table S3. Binning statistic and bins taxonomic annotation.

## Abbreviations

CP: chronic periodontitis. T2DM: type 2 diabetes mellitus. CPT2DM: chronic periodontitis with type 2 diabetes mellitus. IDF: International Diabetes Federation. CoDa: compositional data analysis. GRM: genome-resolved metagenomics. MAG: metagenome-assembled genome. NMDS: non-metric multidimensional scaling. PHP: patient hygiene performance. OHI-S: simplified oral hygiene index. PMA: papillary-marginal-alveolar index. PBI: papillary bleeding index. CPITN: community periodontal index of treatment needs.
